# The effect of time between procedures upon the proficiency gain period for minimally invasive esophagectomy

**DOI:** 10.1007/s00464-019-06692-3

**Published:** 2020-04-20

**Authors:** Sheraz R. Markar, Melody Ni, Hugh Mackenzie, Marta Penna, Omar Faiz, George B. Hanna

**Affiliations:** 1 Division of Surgery, Department Surgery & Cancer, Imperial College London, 10th Floor QEQM Building, London, UK; 2 St Mark’s Hospital and Academic Institute, South Wharf Road, Harrow, London, W2 1NY UK

**Keywords:** Minimally invasive, Esophagectomy, Esophageal cancer, Proficiency gain

## Abstract

**Background:**

Complex surgical procedures including minimally invasive esophagectomy (MIE) are commonly associated with a period of proficiency gain. We aim to study the effect of reduced procedural interval upon the number of cases required to gain proficiency and adverse patient outcomes during this period from MIE.

**Methods:**

All adult patients undergoing MIE for esophageal cancer in England from 2002 to 2012 were identified from Hospital Episode Statistics database. Outcomes evaluated included conversion rate from MIE to open esophagectomy, 30-day re-intervention, 30-day and 90-day mortality. Regression models investigated relationships between procedural interval and the number of cases and clinical outcomes during proficiency gain period.

**Results:**

The MIE dataset comprised of 1696 patents in total, with procedures carried out by 148 surgeons. Thresholds for procedural interval extracted from change-point modeling were found to be 60 days for conversion, 80 days for 30-day re-intervention, 80 days for 30-day mortality and 110 days for 90-day mortality. Procedural interval of MIEs did not influence the number of cases required for proficiency gain. However, reduced MIE procedural interval was associated with significant reductions in conversions (0.16 vs. 0.07; *P* < 0.001), re-interventions (0.15 vs. 0.09; *P* < 0.01), 30-day (0.12 vs. 0.05; *P* < 0.01) and 90-day (0.14 vs. 0.06; *P* < 0.01) mortality during the period of proficiency gain.

**Conclusions:**

This national study has demonstrated that the introduction of MIE is associated with a period of proficiency gain and adverse patient outcomes. The absolute effect of this period of proficiency gain upon patient morbidity and mortality may be reduced by reduced procedural interval of MIE practice within specialized esophageal cancer centers.

**Electronic supplementary material:**

The online version of this article (10.1007/s00464-019-06692-3) contains supplementary material, which is available to authorized users.

Interventional procedures are commonly associated with a period of proficiency gain as the clinician’s experience in performing the new technique grows. Previous institutional and national population-based studies have established measurable adverse effects upon patient outcomes, such as procedure-related mortality, as clinicians obtain technical proficiency [1–4]. Risk-adjusted cumulative sum curve (RA-CUSUM) analysis is a commonly employed statistical methodology to model this period of proficiency gain [2–6]. These graphs plot observed minus the expected outcome (based upon prediction model) against case number in a sequential fashion. Typically at a certain case number, observed is equal to the expected outcome, and the RA-CUSUM curve plateaus and a change-point is reached in the clinician’s learning [7].

Several previous publications have established a measurable relationship between annual procedural volume and clinical outcome from major complex gastrointestinal surgical procedures [8–10]. This body of research has led to health policy changes with the centralization of complex major surgery, such as esophagectomy, to high-volume centers [11, 12]. Further research has assigned greater importance to surgeon volume over hospital volume in determining prognosis from major surgery and specifically esophagectomy [13, 14]. The relationship between procedural interval (i.e., what is the average gap between procedures for an individual surgeon), and number of cases required to gain proficiency has not been previously examined.

The hypothesis under investigation in this national population-based cohort study was that reduced procedural interval will lower the time to reach the plateau in the proficiency gain curve and also reduce adverse outcomes for patients undergoing minimally invasive esophagectomy (MIE) for esophageal cancer during the period of proficiency gain.

## Methods

### Database and coding

Data were derived from the Hospital Episode Statistics (HES) database [15]. All patients over the age of 17 years who underwent elective MIE for esophageal cancer between January 1, 2002, and March 31, 2012, were included in the study. Cancer diagnoses were identified using the relevant International Classification of Disease 10th revision (ICD-10) codes (C15 and D00.1). Procedures were identified using the Office of Population Censuses and Surveys Classification of Surgical Operations and Procedures 4th revision (OPCS) codes. These were G01, G02 and G03 for esophagectomy and were used in combination with Y50.8, Y75, and Y71.4 for laparoscopic and Y49.8 and Y74 for thoracoscopic to identify those patients receiving MIE. Permissions for the comparison of anonymized administrative data were obtained from the National Information Governance Board for Health and Social Care in England.

### Outcomes

Outcome measures evaluated included conversion rate from MIE to open esophagectomy, 30-day re-intervention (surgical), 30-day and 90-day mortality.

### Statistical analysis

We retrieved administrative datasets on MIE resections (OES) (*N* = 1696). To ensure meaningful analyses of the proficiency-gain curves, we selected clinicians who had performed a minimum of 5 cases including at least one adverse event (i.e., conversion, re-intervention or mortality). We also performed a subset analysis of those surgeons who performed a minimum of 20 cases including one adverse event. We analyzed conversion rates, re-intervention, 30-day mortality and 90-day mortality for MIE.

#### Procedural interval (days)

We assessed the *procedural interval* by the ratio of the number of days elapsed since the last case and the number of cases carried out so far. For example, if Surgeon A carried out his first, second and third case (i.e., Case 1, 2 and 3) on Day 1, Day 30 and Day 60, respectively. The *procedural intervals* were, respectively, 0 (= 0/1) for Case 1, 15 days (= 30/2) for Case 2 and 20 days (= 60/3) for Case 3.

#### Relationship between procedural interval and clinical outcomes

We performed logistic regressions to investigate relationships between procedural interval and the clinical outcomes, adjusting for patient age (< 70 years or ≥ 70 years), sex (male or female) and medical comorbidities as measured by the Charlson comorbidity index score (< 2 or ≥ 2). To understand potential differences from procedural intervals that were small vs. large, we defined low procedural interval cases as those cases carried out within certain time limit from the previous cases, e.g., within 7 days. To determine what these limits are, we carried out change-point modeling whereby multivariate logistic regression models were fitted for each of the outcome variables using patient age, sex and Charlson scores [16] as independent variables. Procedural interval was captured by a dummy variable that took the value of 1 or 0, which corresponds to whether or not the case gap was below or above the cutoff value. Different relationships between an outcome variable and procedural interval were captured by the interaction between the dummy variable and the patient comorbidities.

We assumed that such cutoffs might take any value between the minimum and maximum procedural interval. We ran the change-point models iteratively, using a gap of 5 between the cutoffs. That is, if the minimum and maximum procedural interval was 0 and 100, we ran the model under assumed cutoffs of 5, 10, 15,…, 100. We then compared the model fit and derived the cutoff from the model that had the smallest deviances.

These cutoffs were used to define low procedural interval cases. We also carried out multivariate logistic regressions to investigate relationships between procedural interval and the clinical outcomes.

#### Relationship between procedural interval and number of cases required to gain proficiency

We analyzed number of cases required to gain proficiency for each individual surgeon also by employing change-point modeling. One modification was the definition of dummy variable, which took the value of 1 or 0 depending on whether the *case volume* was above or below certain cutoffs. We assumed that a cutoff could take any value from 1 to the maximum. The number of cases required to gain proficiency was derived from the best-fitted regression model (as above) for each individual surgeon. We then carried out a linear regression to investigate the relationship between the number of cases required to gain proficiency and average procedural interval by surgeon within their respective proficiency gain period.

We used *t* test for continuous variables and Chi-square test for categorical variables to identify any significant differences between age, sex and Charlson comorbidity index score and case gaps. We performed the analyses in Excel and in open-source software R (version 3.3.0 for Mac OS X, generalized linear mixed models package lme4).

## Results

### Data

The MIE dataset comprised of 1696 patents in total, with procedures carried out by 148 surgeons. The mean conversion, 30-day re-intervention, 30-day and 90-day mortality rates were 5.43%, 8.43%, 3.42% and 5.31%, respectively. Logistic regression analysis found that patient age was a significant predictor of 30-day mortality (*P* < 0.05). No other patient factors (sex, Charlson) reached statistical significance for any other patient outcomes (Online Appendix A).

### Relationship between procedural interval and clinical outcomes

**Fig. 1:**
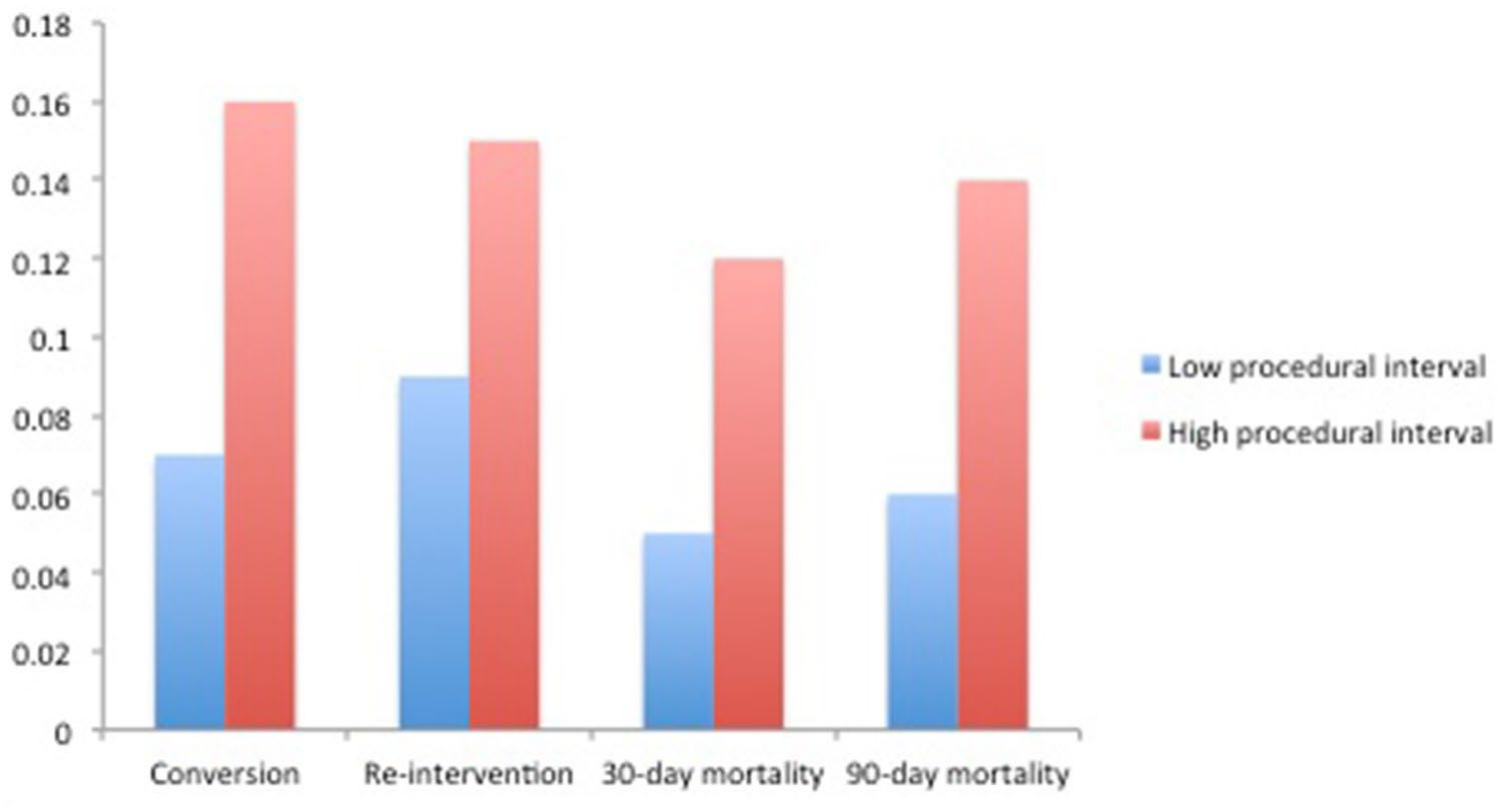
Mean outcomes for low vs. high procedural interval of practice

Table 1 shows output from logistic regression models where procedural interval served as a continuous independent variable for each of the four outcome variables. Thresholds extracted from procedural interval change-point modeling were found to be 60 days for conversion, 80 days for 30-day re-intervention, 80 days for 30-day mortality and 110 days for 90-day mortality. As shown, for each of the four outcomes, the mean conversion, re-intervention, 30-day and 90-day mortality rates were significantly reduced with reduced compared to increased procedural intervals, which were performed within the threshold (Fig. 1).

**Table 1:** Impact of procedural interval and mean values at low vs. high procedural interval cases

Outcome	Effect of procedural interval (exp(B))	Mean and range of procedural intervals	Threshold (days)	Mean at low procedural interval (± sd)	Mean at high procedural interval (± sd)
Conversion	1.006***	64 (0–357)	60	0.07 (0.26)	0.16 (0.37)***
Re-intervention	1.004***	67 (0–619)	80	0.09 (0.28)	0.15 (0.36)**
30-day mortality	1.006***	58 (0–545)	80	0.05 (0.21)	0.12 (0.33)**
90-day mortality	1.004***	61 (0–609)	110	0.06 (0.24)	0.14 (0.35)**

^#^n.s

*P value < .05; **P value < .01; ***P value < .001

Subset analysis was performed for surgeons who performed 20 or more cases, with a similar finding of the mean conversion, re-intervention, 30-day and 90-day mortality rates were significantly reduced with reduced compared to increased procedural intervals (Online Appendix B, Supplementary Table 1).

### Relationship between procedural interval and number of cases required to gain proficiency

**Fig. 2:**
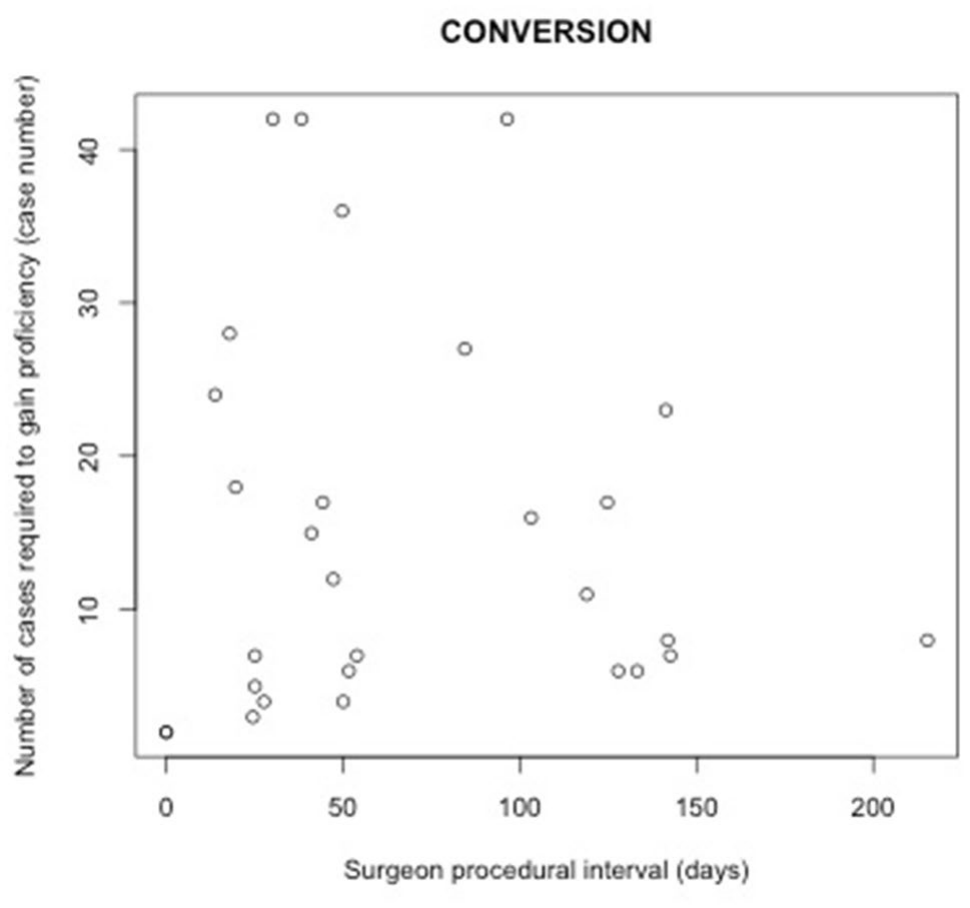
Relationship between procedural interval and number of cases required to gain proficiency measured by conversion for MIE

**Fig. 3:**
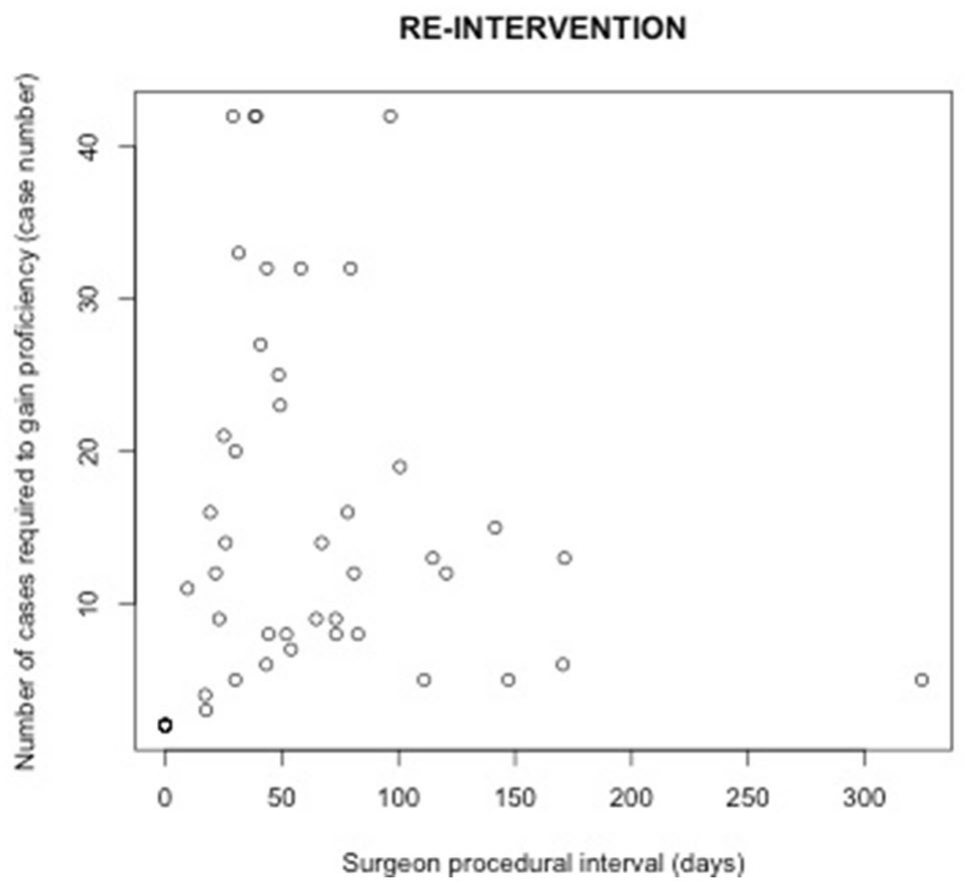
Relationship between procedural interval and number of cases required to gain proficiency measured by 30-day re-intervention for MIE

**Fig. 4:**
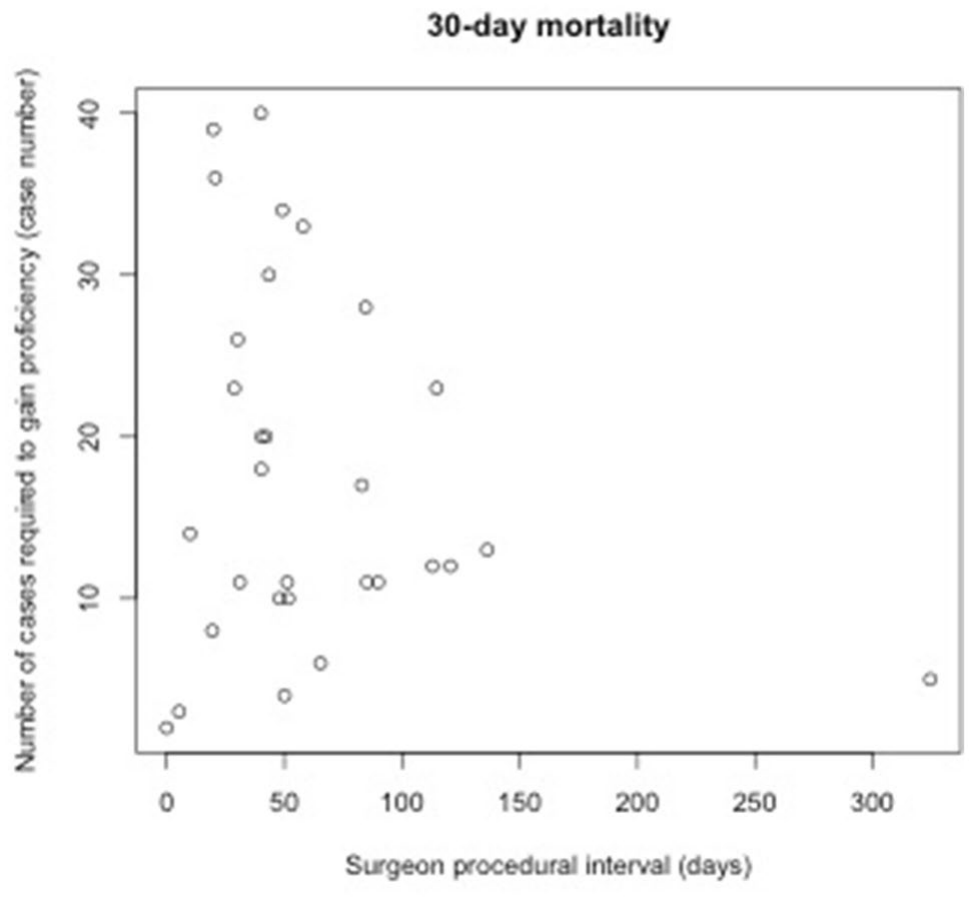
Relationship between procedural interval and number of cases required to gain proficiency measured by 30-day mortality for MIE

**Fig. 5:**
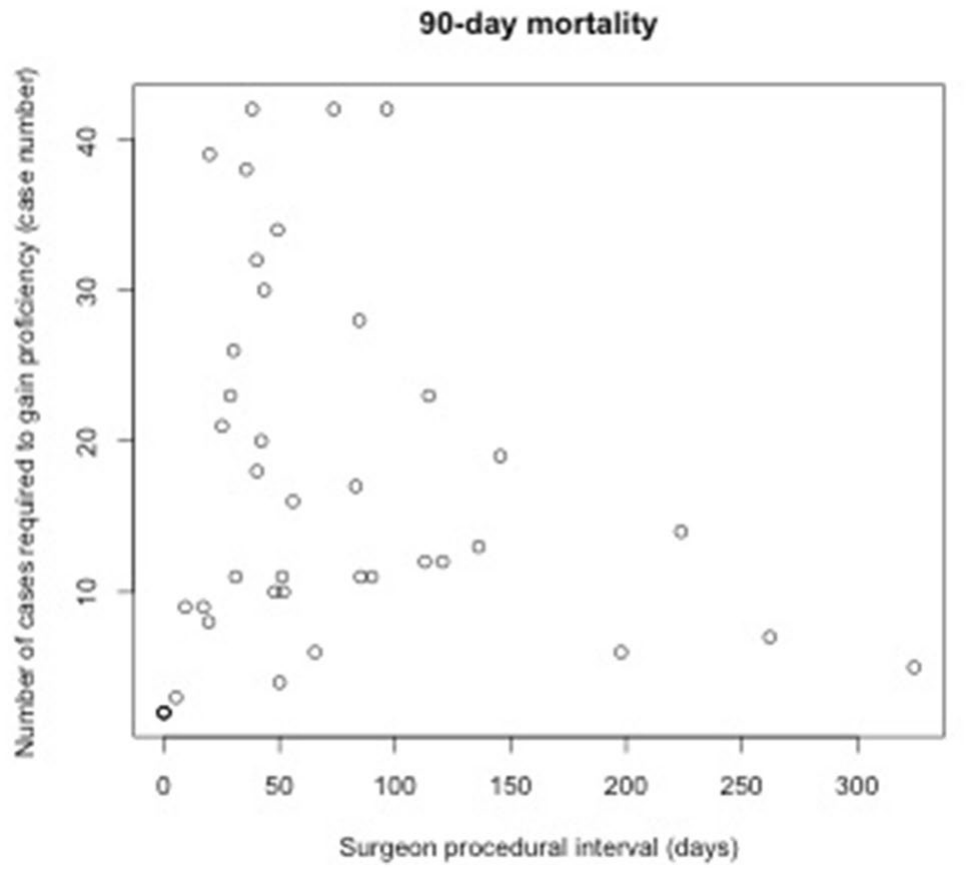
Relationship between procedural interval and number of cases required to gain proficiency measured by 90-day mortality for MIE

Table 2 shows the result of an analysis examining relationships between procedural intervals and the number of cases required to gain proficiency at the individual surgeon level. Such relationships were statistically insignificant across all four outcomes for MIE (Figs. 2, 3, 4, 5). Subset analysis was performed for surgeons who performed 20 or more cases, with a similar finding of no statistical significance seen across all found outcomes for MIE (Online Appendix B, Supplementary Table 2).

**Table 2:** Relationship between the number of cases required to gain proficiency and surgeon procedural interval

Endpoints	# of surgeons	Mean length (range) of learning curves by surgeon in cases	Coefficient of procedural interval: mean (sd)	*P* value
Conversion	29	15 (2–42)	− 0.016 (0.045)	0.73
Re-intervention	52	14 (2–42)	0.013 (0.029)	0.65
30-day mortality	30	18 (2–40)	− 0.041 (0.034)	0.24
90-day mortality	41	17 (2–42)	− 0.018 (0.027)	0.52

## Discussion

This national population-based cohort study for minimally invasive esophagectomy suggests that procedural interval between MIEs does not influence the number of cases required to gain proficiency. However, reduced procedural interval was associated with reductions in adverse patients outcomes, specifically fewer conversions and re-interventions, and less 30-day and 90-day mortality during the period of proficiency gain.

The population-based design with virtually complete inclusion of all eligible patients in England is a strength of this study. The large sample size, complete follow-up of all patients, and the adjustment for several relevant confounding factors are other advantages. Given the nature of the national database and the retrospective design used for the study, it was not possible to define patient clinical drivers that may have influenced surgical treatment allocation. It must be further acknowledged as a limitation that from a national administrative dataset such as HES used in this analysis we cannot identify individual hospital related factors, including size of the unit, number of surgeons or number of ITU beds, which may have contributed to the improvements in mortality observed with reduced surgeon procedural interval. Furthermore, administrative datasets such as HES do not provide data regarding surgical parameters such as operative time or intraoperative blood loss, as well as cause of death, and the data presented is for all-cause mortality. As a large national database study the results generated are dependent upon the reliability of the methodology and accuracy of data collection, which is a limitation shared by all national administrative databases.

Previous publications have established, during the period of proficiency gain, a measurable increase in adverse short- and long-term mortality from minimally invasive and open esophagectomy [4, 17]. Furthermore, similar adverse effects during this ‘learning period’ for less invasive procedures such as endoscopic mucosal resection for esophageal cancer have also been demonstrated [2]. However, a recent single institutional study by Phillips et al. [18] was able to demonstrate within a high-volume center that patient outcomes are not compromised by supervised trainee involvement in transthoracic esophagectomy. The results of the present study suggest that adverse outcomes associated with the period of proficiency gain in performing a new technique such as MIE can be reduced when conducted with reduced procedural intervals, despite a similar number of cases required to gain proficiency. Surgeons practicing with reduced procedural intervals are more likely to be practicing within esophageal surgery centers of excellence. Thus, the reduced mortality during the proficiency gain period may be a reflection of strategies to rescue following complications from MIE and improvements in multi-disciplinary care seen within esophageal surgery centers.

Specifically, in England since 2001, following a national policy recommendation, there has been a consistent shift toward the centralization of esophageal and gastric cancer resections to high-volume centers, with subsequent consistent improvement in perioperative mortality [12]. Previous publications have established that high hospital volume centers are more likely to re-intervene successfully and prevent mortality following major gastrointestinal surgery [10, 19]. High-volume centers may be better set up to successfully manage complications during the period of proficiency gain for complex procedures. Likely reasons for this include 24-h access to endoscopy, highly experienced interventional radiology, intensive care and specialist medical services and thus reduces the adverse effects experienced by patients during a surgeon’s period of proficiency gain.

In conclusion, this national population-based cohort study has demonstrated that the introduction of MIE is associated with a period of proficiency gain and adverse patient outcomes. The absolute effect of this period of proficiency gain upon patient morbidity and mortality can be reduced by lower procedural intervals within specialized esophageal cancer centers, capable of managing complications with strategies for rescue from mortality. This study shows further benefits for the centralization of esophageal surgery.

## Electronic supplementary material

Below is the link to the electronic supplementary material.

Supplementary material 1 (DOCX 65 KB)

Supplementary material 2 (DOCX 70 KB)
