# Supplementary material for: The effect of time between procedures upon the proficiency gain period for minimally invasive esophagectomy
Source: Surg Endosc. 2020 Apr 20;34(6):2703–8. doi: 10.1007/s00464-019-06692-3 (PMC7214481; doi:10.1007/s00464-019-06692-3)
Supplement: Supplementary file 1 — Supplementary material 1 (DOCX 65 KB) [file 464_2019_6692_MOESM1_ESM.docx]

**Appendix A**

| 30 day mortality |  | Estimate | Std. | p value |
| --- | --- | --- | --- | --- |
|  | (Intercept) | -4.85 | 1.14 | 0.000 |
|  | startage | 0.03 | 0.02 | 0.037 |
|  | sex | -0.54 | 0.37 | 0.145 |
|  | Charlson | 0.01 | 0.05 | 0.862 |
|  |  |  |  |  |
| 90 day mortality | (Intercept) | -3.87 | 0.90 | 0.000 |
|  | startage | 0.02 | 0.01 | 0.080 |
|  | sex | -0.45 | 0.29 | 0.123 |
|  | Charlson | 0.04 | 0.04 | 0.390 |
|  |  |  |  |  |
| Reintervention | (Intercept) | -2.71 | 0.68 | 0.000 |
|  | startage | 0.01 | 0.01 | 0.414 |
|  | sex | 0.07 | 0.20 | 0.728 |
|  | Charlson | -0.08 | 0.04 | 0.063 |
|  |  |  |  |  |
| Conversion | (Intercept) | -4.63 | 0.87 | 0.000 |
|  | startage | 0.02 | 0.01 | 0.089 |
|  | sex | 0.32 | 0.24 | 0.181 |
|  | Charlson | 0.01 | 0.04 | 0.765 |
