# Supplementary material for: The effect of time between procedures upon the proficiency gain period for minimally invasive esophagectomy
Source: Surg Endosc. 2020 Apr 20;34(6):2703–8. doi: 10.1007/s00464-019-06692-3 (PMC7214481; doi:10.1007/s00464-019-06692-3)
Supplement: Supplementary file 2 — Supplementary material 2 (DOCX 70 KB) [file 464_2019_6692_MOESM2_ESM.docx]

**Subset analysis of surgeons who performed a minimum of 20 cases including one adverse event.**

**Supplementary Table 1. Impact of procedural interval and mean values at low vs. high procedural interval cases**

| Outcome | Effect of procedural interval  (exp(B)) | Mean and range of procedural intervals | Threshold | Mean at low procedural interval (±sd) | Mean at high procedural interval (±sd) |
| --- | --- | --- | --- | --- | --- |
| Conversion | 1.007** | 58 (0-341) | 190 days | 0.09 (0.28) | 0.33 (0.50) * |
| Re-intervention | 1.005* | 55 (0-402) | 50 days | 0.06 (0.24) | 0.12 (0.33) * |
| 30-day mortality | 1.004* | 50 (0-341) | 80 days | 0.04 (0.20) | 0.09 (0.29) * |
| 90-day mortality | 0.998 | 52 (0-402) | 110 days | 0.06 (0.23) | 0.12 (0.32) * |

^#^n.s. **p* value<.05; ***p* value <.01; ****p* value <.001

**Subset analysis of surgeons who performed a minimum of 20 cases including one adverse event.**

**Supplementary Table 2. Relationship between the number of cases required to gain proficiency and surgeon procedural interval**

| Endpoints | # of surgeons | Mean length (range) of learning curves by surgeon in cases | Coefficient of procedural interval: mean(sd) | p value |
| --- | --- | --- | --- | --- |
| Conversion | 20 | 19 (2-42) | -0.014 (0.068) | 0.84 |
| Re-intervention | 29 | 19 (2-42) | -0.011  (0.058) | 0.84 |
| 30-day mortality | 19 | 21 (2-40) | 0.024  (0.088) | 0.79 |
| 90-day mortality | 27 | 20 (2-42) | 0.012  (0.066) | 0.09 |
